# The devil effect triggered by sexual crimes

**DOI:** 10.3389/fpsyg.2025.1602972

**Published:** 2025-08-18

**Authors:** Michaela Pfundmair, Romana Matanovic

**Affiliations:** Faculty of Psychology, Alpen-Adria University of Klagenfurt, Klagenfurt, Austria

**Keywords:** halo effect, devil effect, schemas, judgments, sexual crimes

## Abstract

Previous research has identified a number of stereotypical beliefs about sexual crimes, particularly in relation to child sexual abuse and rape. We suggested that these beliefs may be the result of a negative halo effect (i.e., a single negative attribute biases subsequent impression formation judgments). We therefore hypothesized that mere keyword pairs containing ‘sex’ activate negative schemas that influence judgments of criminal cases. We conducted three studies to investigate this hypothesis. In a focus group interview, we attempted to gain a basic understanding of the hypothesized effect. Two online surveys were used to quantify the initial findings. The results showed that the keywords ‘sex and children’ triggered strong negative schemas such as acts of violence, pedophile offenders, and a desire for harsh punishments. The keywords ‘sex and violence’ activated impressions of a broader range of offenses, but still strongly negative associations about the possible offender and harsh penalties. In contrast, the combined keywords ‘children and violence,’ which served as a control, elicited more heterogeneous responses. Overall, the current findings confirm the idea of a devil effect triggered by sexual crimes. This effect could have serious consequences, from reduced awareness of actual crimes to biased judgments by judges and juries.

## 1 Introduction

Imagine reading a newspaper article with the headline: ‘Sex and children: Court verdict eagerly awaited.’ Would you expect to read about a gruesome case of a pedophile child sex offender rather than an intimate relationship between two minors? An answer to these questions might be given by Asch (1946, p. 258) who stated: ‘A glance, a few spoken words are sufficient to tell us a story about a highly complex matter.’ With this quote, Asch depicted very early on in the history of psychology that people quickly form impressions even if they do not have enough information to draw a reliable conclusion. Models developed on the basis of such findings highlighted that people are subject to category-based processes by default to form impressions of others. The Continuum Model, for example, assumed that people react quickly, captured by schema-triggered affects, stereotypical associations, and discriminatory responses (Fiske and Neuberg, 1990). The current work examines such quick impressions using a small information base and investigates whether they are systematic in sexual crimes. More specifically, we investigate whether people are subject to a so-called devil effect as soon as they have clues for a sex offense.

### 1.1 Stereotypical beliefs about sexual crimes

Previous research has identified a number of stereotypical beliefs—so called rape myths—about individuals convicted of child sexual abuse: Many people believe that they are strangers (Kernsmith et al., 2009; McCartan et al., 2015) or ‘dirty old men’ who use violence, aggression or threats in committing sexual offenses against children (Fuselier et al., 2002). It is therefore unsurprising that many people advocate for post-incarceration policies for offenders who assault child victims (Pickett et al., 2013; Rogers et al., 2011). However, people convicted of child sexual abuse are usually family members or persons in positions of trust who do not use physical violence (Lieb et al., 1998; Loinaz Calvo et al., 2019). Moreover, pedophilia (the primary or exclusive sexual interest in prepubescent children; APA, 2013) is often equated and confused with sexual abuse of a child (Harper et al., 2018). As result, people have strong negative emotional reactions to pedophiles and advocate harsher punishments, including death, even for non-offending pedophiles (e.g., Imhoff, 2015; Jahnke et al., 2015a; Maroño and Bartels, 2020). However, the reality is that not every case of child sexual abuse is motivated by pedophilia and not every person with pedophilia commits sexual assaults. Individuals convicted of child sexual abuse are only classified as pedophiles in 45–50% of cases; all other cases are labeled as substitute offenders (Seto, 2009). The latter have no genuine sexual interest in children, but molest them for a number of situational reasons, e.g., stress or availability of the children (Robertiello and Terry, 2007).

There are also stereotypical beliefs about the rape of adults. The so-called ‘real-rape’ stereotype involves a female victim who is violently assaulted by a stranger at night (Horvath and Brown, 2009). Another myth about perpetrators of rape is that they are mentally ill (Groth and Birnbaum, 2013). These stereotypical beliefs even seem to carry over into the criminal justice system: The more a situation deviates from this ‘real rape,’ the less likely is it to be investigated by the police, prosecuted by the justice system, or result in a conviction (Ellison and Munro, 2013; Willmott and Hudspith, 2024). Although this stereotype is widely held in society, it differs from reality in most cases. In reality, rape is most likely committed by a partner or acquaintance, often in alcohol-fueled situations (Kelly et al., 2005), and is accompanied by widely varying forms or degrees of physical resistance or injury by the victim (Gray-Eurom et al., 2002). Moreover, only a small proportion of sex offenders suffer from mental or sexual preference disorders (Sarkar, 2013).

In a nutshell, people seem to have preconceived opinions about sexual offending. However, these stigmatizing judgments about sex offending may not just be stereotypes (i.e., schemas applied to members of a social group). Previous research has suggested that they could be the result of a specific halo effect (Jahnke, 2018; Maroño and Bartels, 2020), in which a single characteristic leads to unwarranted conclusions (Forgas and Laham, 2009). The aim of this work is to test this assumption for the first time.

### 1.2 The halo and the devil effect

The ‘halo effect,’ originally proposed by Thorndike (1920), is an important effect in impression formation and a form of heuristic processing. It is the tendency to assume that once a person has some known characteristics, their other, unknown (and also unrelated) characteristics are likely to match. Thorndike (1920) referred to the phenomenon as the ‘devil or horns effect’ when it relates to negative characteristics. That is, a single negative attribute biases subsequent impression formation judgments on unknown dimensions, but in a negative direction. Ultimately, the halo and the devil effect are constructive cognitive illusions characterized by a general confirmation bias (Forgas and Laham, 2017).

In early research, these effects were demonstrated in a study in which participants were asked to watch a video tape of a professor’s lecture. If he was initially portrayed as friendly, participants had a positive impression about him; if he was initially portrayed as arrogant, his ratings dropped (Kelley, 1950). The most intensely researched halo effect is the ‘what is beautiful is good’ effect. This means that people associate positive attributes with attractiveness, for example, higher intelligence, kindness, and honesty (Dion et al., 1972). In addition to attractiveness, other attributes can also distort impression formation. For example, a person’s name: In one study, a poem and a painting signed with an unusual name were judged to be more creative than the same works created by a person with a conventional name (Lebuda and Karwowski, 2013). In the context of jury decision making, impression formation is, for example, biased by ethnicity and socioeconomic status, such that defendants are at a disadvantage if they are Mexican Americans and of low socioeconomic status (Esqueda et al., 2008).

Considering the known preconceived notions about criminal acts involving sex, it is reasonable to assume that it is not just stereotypes that distort impressions about sexual offenses, but a devil effect. That is, little information about a case could activate a negative schema about the associated crime that negatively influences further judgments about that case.

### 1.3 The devil effect triggered by sexual crimes: the current work

Based on the outlined considerations, we expected in the present work that keywords containing ‘sex’ would evoke a devil effect, i.e., an overall impression of a criminal case including a specific offense, a specific offender, and, related to this, a specific punishment perceived as just. More specifically, we hypothesized that the combination of the keywords ‘sex and children’ would activate an extremely negative schema of a criminal case involving a violent and pedophilic offender and a desire for harsh punishments. Moreover, we predicted that the combination of the keywords ‘sex and violence’ would trigger similarly extreme impressions, including a violent and mentally ill offender and a desire for maximum penalties. To be clear, sexual crimes can present themselves as the devil effect would predict. But they can also look quite different. The limited view that they present only or primarily as the former will be investigated in the current work. As a control condition, we used the combined keywords ‘children and violence.’ This combination of words is similarly negatively as the critical keywords. However, since it is not associated with specific stereotypes, we did not expect a devil effect. Instead, we predicted that these keywords would trigger rather heterogeneous associations. Thus, they served as a control for our predictions.

To investigate our hypotheses, we followed a multimethodological approach, combining a qualitative interview (Pilot Study) with quantitative research (Studies 1 and 2). In the pilot and first study, we tested the effect of mere keywords on people’s impression formation about criminal cases to explore whether it only takes a little information to trigger certain negative schemas related to sexual crimes. In the second study, we investigated whether the activated schemas influenced judgments about a particular criminal case. Importantly, in the current work, we explicitly wanted to examine a devil effect and not stereotypical beliefs. The latter arise when an observer believes that all individuals in a given group are the same. Accordingly, in studies investigating stereotypes, participants are usually instructed to express their opinion about a social group (e.g., Schneider and Bos, 2014). Halo or devil effects, on the other hand, occur when the observer focusses on one attribute and then assigns further attributes to this person on the basis of previous attributes. Accordingly, halo effects are measured by providing one trait and asking for the likelihood of other traits (e.g., Gräf and Unkelbach, 2016). All current studies focused on the latter. Consequently, instead of presenting a specific group, participants in the current studies were presented with the critical keywords and then judged the likelihood of several attributes.

Materials and data of all studies can be accessed at https://doi.org/10.17605/OSF.IO/MUQTH.

## 2 Pilot study

The pilot study served to gain initial insights into whether a small amount of information is sufficient to trigger certain negative schemas in connection with sexual offenses. To create open response options, participants were asked to freely discuss which offenses, offenders, and punishments they associated with the critical keywords. We opted for a focus group interview, in which several participants are interviewed at the same time. In this way, the attitudes and emotions of both individuals and the entire group can be explored, benefitting from mutual stimulation (Aghamanoukjan et al., 2009). To avoid a possible ceiling effect, we chose a mixed sample of professionals and laypeople, as individuals who lack relevant knowledge are particularly likely to hold schema-based beliefs (e.g., Sanghara and Wilson, 2006).

We hypothesized that the combination of the keywords ‘sex and children’ would activate an extremely negative schema of a criminal case involving impressions of violence, pedophilia, and harsh punishments. Similarly, we expected that the combined keywords ‘sex and violence’ would evoke the impression of a violent and mentally ill offender as well as maximum penalties. We did not expect any systematic responses for the combined keywords ‘children and violence.’

### 2.1 Method

#### 2.1.1 Participants

We adhered to the number of participants of six to eight persons to conduct a focus group interview recommended by Krueger and Casey (2000). Thus, the sample consisted of seven participants (4 female, 3 male; *M* = 32.86 years, *SD* = 8.84) who volunteered to participate after the study was announced at an Austrian university and at a large Austrian healthcare company specializing in the mental health of clients who have a criminal record or are undergoing rehabilitation. Four participants were students of different study programs. These were our laypersons. The other three were professionals working for the healthcare company.

#### 2.1.2 Procedure and materials

After informed consent was obtained, the participants were seated in a circle and the research assistant switched on the video recording. Following a prepared interview guideline, the research assistant moderated the group discussion. The research assistant made sure that each participant spoke to each keyword and that the discussion developed a natural flow. The participants’ discussion lasted a total of 2 h, including a short restroom break during which they should not talk about the subject of the study. At the end, participants were thanked and debriefed in detail. The study was conducted in German. It was approved by the Ethics Council of the Institute of Psychology at the University of Klagenfurt (ER-PSY; ethics ID reference number: 2018–087).

##### 2.1.2.1 Interview guideline

The research assistant semi-structured the interview using the following questions: ‘What do you think when you hear the words [keywords]? (A) What criminal offense do you associate with it? (B) Which offender do you associate with it? (C) What punishment would you consider appropriate?’ Notably, participants were asked to make free associations with the keywords before we asked them to answer the three specific questions. No options were given for the latter either; participants answered freely. After the group had discussed the first keyword pair (sex and violence), the research assistant presented the second (sex and children) and third keyword pair (children and violence) in turn.

##### 2.1.2.2 Analysis strategy

The video recording was fully transcribed by the research assistant for the content analysis. (The full transcription can be found in the Online Supplementary: https://doi.org/10.17605/OSF.IO/MUQTH). This transcription was then analyzed according to the procedures of qualitative content analysis (Mayring, 2010) using QCAmap (Fenzl and Mayring, 2017). This approach follows a step-by-step processing of the text material. Once categories have been defined, the material is assigned to these categories. We opted for a deductive approach in which the coding guideline was defined *a priori*. Text passages were assigned to the three categories (1) offense, (2) offender and (3) punishment for each keyword pair as soon as they matched the categories.

### 2.2 Results

In the following, all of the participants’ mentions that could be assigned to the predefined categories are presented. In addition, the total number of mentions per category is reported. If a mention occurred more frequently, the number of similar mentions is also displayed in parenthesis. This indicates whether the participants had systematic schemas in relation to the three keywords or not.

Child pornography (mentioned 2 times) and rape lasting for years with the keyword pair. Participants characterized possible offenders (total: 10 mentions) as abnormal (2 times), (highly) ill, (2 times) and psychopathic. They also pointed out that such offenders are socially capable and outwardly normal. Moreover participants mentioned the term pedophilia as well as power motives and that the offenders did not get along with women. As just punishments (total: 12 mentions) the participants strongly advocated a life sentence (4 times) and that the offender should be locked away and removed from society. They also discussed illegal punishments such as wearing a discriminatory tattoo genital mutilation pain and death; additionally they described vigilante justice as just. Only one participant felt that none of the punishments proposed by the other participants were appropriate and suggested a hiking project for therapy.

*Keyword pair ‘sex and violence.’* As offenses (total: 3 mentions) participants associated rape and (sexual) abuse with the keyword pair whereby they also stated that repeated offenses were particularly likely. They characterized the offenders (total: 3 mentions) as perverted sick and victims themselves. They spoke out in favor of life imprisonment (4 times) or at least 10 years in prison as appropriate punishments (total: 8 mentions) with 6 years being too little. They also called for the public to be given access to the offender’s criminal record (in contrast to the current law). Only one participant considered that imprisonment of offenders to be pointless and suggested psychological processing of the offense with the help of a mentor.

*Keyword pair ‘children and violence.’* Participants associated both psychological and physical violence the latter in the form of beatings and (less severe) slaps with the keyword pair (total: 3 mentions). As offenders (total: 6 mentions) they named overburdened weak and impatient parents with communication difficulties and low tolerance. Participants also named narcissistic personalities. When asked about just punishments (total: 8 mentions) supportive suggestions in the form of family help parenting therapy (3 times) and preventive suggestions in the form of a ‘parenting license,’ and a law stating that not everyone is allowed to have children were mentioned. Only in the case of severe physical harm did they suggest taking a child from the family (2 times).

### 2.3 Discussion

The Pilot Study indicated two points: Firstly, people seem to have clear ideas about sexual crimes, even if they only have a very limited information base about the latter. Two terms were sufficient to spin together a specific criminal offense. Secondly, these impressions seem to follow a systematic pattern: The combination of the keywords ‘sex and children’ activated an extremely negative schema of criminal cases that included violent acts (associations of rape), sick and pedophile offenders, and a desire for harsh punishments that go beyond legality. The combination of the keywords ‘sex and violence’ elicited a broader range of impressions of possible offenses (both rape and, despite the violence keyword, abuse) and thus somewhat more balanced associations, but still highly negative impressions of possible offenders (being perverse and sick) and maximum penalties. Only one participant felt that the punishments proposed by the interview partners were too extreme. The Pilot Study thus suggests that two attributes are sufficient to activate certain negative schemas in the case of sexual offenses. A different pattern emerged for the keywords ‘children and violence.’ Here, the participants named a whole range of possible offenses (from slapping to beating), offenders (from overburdened parents to narcissist persons) and punishments (from support to mild penalties).

The Pilot Study investigated sexual crime schemas in an open-ended process. However, due to the small number of participants, the findings are clearly not representative. Moreover, the methodological approach was uncontrolled: The keyword pairs were presented in a specific order, and the group discussion, which was part of the concept of the focus group interview, could also have influenced the individual responses of the participants. Thus, biases such as contrast or conformity effects cannot be ruled out. However, it should be borne in mind that the Pilot Study only served as a trial balloon to examine the possible existence of a devil effect in a random sample. The following studies served to investigate this effect using more reliable methodological approaches.

## 3 Study 1

In the Pilot Study, we found initial evidence of negative schemas associated with sexual crimes. In Study 1, we aimed to investigate the devil effect using an improved methodological approach. Participants were presented with the critical keywords in randomized order and asked to imagine plausible offenses, offender traits, and punishments. Similar to the Pilot Study, we recruited a more informed sample to avoid ceiling effects on schema-based beliefs that typically occur in individuals who lack relevant knowledge (e.g., Sanghara and Wilson, 2006). Thus, we investigated law students who were familiar with a wide range of criminal offenses. Since these participants were trained in criminal terminology, we decided to ask about possible offenses and offenders in an open text field to avoid being influenced by predefined items. More specific variables related to psychopathy and punishment, which might not have been indicated by the participants in a spontaneous (open) response, were assessed with validated scales. With regard to the offender personality, we specifically investigated the so called ‘Dark Triad,’ a constellation of three socially undesirable but independent personality traits (Paulhus and Williams, 2002): narcissism (characterized by grandiosity and egotism, among other traits), psychopathy (characterized, inter alia, by antisocial behavior and remorselessness), and Machiavellianism (characterized, inter alia, by manipulation and exploitation of others).

We hypothesized that the combined keywords ‘sex and children’ would activate a schema of a criminal case involving violence, pedophilia, and harsh punishments. Similarly, we expected that the combination of the keywords ‘sex and violence’ would trigger impressions of violent and mentally ill offender as well as maximum penalties. We did not expect any systematic reactions to the combined keywords ‘children and violence.’

### 3.1 Method

#### 3.1.1 Participants and design

After excluding participants who had indicated a field of study other than law, our final sample consisted of 62 law students (44 female, 18 male; *M* = 23.19 years, *SD* = 3.57) from German and Austrian universities who voluntarily participated in this study after being recruited via social media. Of these, 27.4% had already completed a court internship and 72.6% had no previous experience.

The study followed a within-subjects design in which the three keyword pairs (sex and children vs. sex and violence vs. children and violence) varied; the variables on the imagined offenses, offenders, and punishments served as dependent variables.

#### 3.1.2 Procedure and materials

The study was conducted in the form of an online survey. After obtaining informed consent, participants were instructed that they would be presented with pairs of words and asked to think about the associated crime without further information. They were then shown one of the three keyword pairs (‘sex and children,’ ‘sex and violence,’ ‘children and violence’); the order of the keyword pairs was randomized between participants to avoid an order effect. Participants were asked to describe their imaginations of the offense and the offender. Moreover, they were asked to answer a scale on the personality of the offender (Dirty Dozen scale, see below) and on the punishment they felt was appropriate. They were then presented with the other two keyword pairs in turn and asked to complete the same items. At the end, participants were thanked and debriefed. Since the study was conducted online, special emphasis was placed on protecting potentially vulnerable individuals. For this reason, the debriefing included an invitation to discuss any questions or suggestions with the principal investigator, as well as a number of addresses for psychological support in case the participants had experienced any stress after the study. The study was conducted in German. It was approved by the Ethics Council of the Institute of Psychology at the University of Klagenfurt (ER-PSY; ethics ID reference number: 2018–042).

##### 3.1.2.1 Offense

In an open text field, participants were asked to briefly describe the crime they were imagining. For example, one participant’s answer was: ‘A middle-aged man rapes a small child.’ These answers were coded by two blind raters to determine the extent to which they matched specific categories. Specifically, the raters rated the participants’ responses using the following eight categories on a coding scale ranging from 1 = *does not match at all* to 5 = *absolutely matches*: rape (inter-rater reliabilities: ICC_sex and children_ = 0.45, ICC_sex and violence_ = 0.67, ICC_children and violence_ = 0.55), child sexual abuse (ICC_SC_ = 0.55, ICC_SV_ = 0.16, ICC_CV_ = 0.80), coercion/harassment/insult (ICC_SC_ = 0.27, ICC_SV_ = 0.38, ICC_CV_ = 0.52), physical violence (ICC_SC_ = 0.34, ICC_SV_ = 0.31, ICC_CV_ = 0.80), psychological violence (ICC_SC_ = 0.29, ICC_SV_ = 0.03, ICC_CV_ = 0.36), forced prostitution (ICC_SC_ = 0.50, ICC_SV_ = 0.53, ICC_CV_ = 0.27), child abduction (ICC_SC_ = 0.39, ICC_SV_ = −0.27, ICC_CV_ = 0.58), consensual sexual activity (ICC_SC_ = 0.75, ICC_SV_ = 1.00, ICC_CV_ = 1.00; notably, we also assessed consensual sexual activities, as these may also constitute a crime under German and Austrian law, depending on the age of the individuals involved [e.g., if they are 12 and 14 years old]). According to Cicchetti (1994), reliability coefficients below 0.40 are considered poor, between 0.40 and 0.59 fair, between 0.60 and 0.74 good, and above 0.75 excellent. However, lower inter-rater reliabilities can also be valid as long as sufficient measurements are available (Gwet, 2014), which was the case here. Thus, we decided to take a less conservative approach and accept ICCs of above 0.30 as sufficient. Variables below this value were excluded from further analyses due to insufficient inter-rater reliabilities (see Table 1 for included and excluded variables).

**Table 1:** Descriptive statistics and results of one sample t-tests comparing the sample mean to the respective middle scale points (Study 1).

	Sex and children			Sex and violence			Children and violence		
	M (SD)	t	Cohen’s d	M (SD)	t	Cohen’s d	M (SD)	t	Cohen’s d
Offense									
Rape	3.22 (1.02)	1.63	0.22	**4.68 (0.75)**	17.13***	**2.25**	1.99 (1.03)	−7.54***	0.98
Child sexual abuse	**4.36 (1.08)**	9.46***	**1.25**	–	–	–	2.80 (1.61)	−0.97	0.13
Coercion/harassment/insult	–	–	–	**3.66 (0.86)**	5.77***	**0.76**	2.41 (1.04)	−4.36***	0.57
Physical violence	**3.52 (0.98)**	4.00***	**0.53**	**3.50 (0.88)**	4.31***	**0.57**	**4.69 (0.81)**	15.98***	**2.08**
Psychological violence	–	–	–	–	–	–	**3.92 (0.97)**	7.29***	**0.95**
Forced prostitution	1.76 (0.83)	−11.25***	1.49	1.98 (0.86)	−8.97***	1.18	–	–	–
Child abduction	1.61 (0.67)	−15.65***	2.07	–	–	–	1.39 (0.66)	−18.64***	2.43
Consensual sexual activity	1.11 (0.59)	−24.32***	3.22	1.00 (0.00)	0.00	0.00	1.00 (0.00)	0.00	0.00
Offender									
Pedophile	**3.24 (0.71)**	2.53*	**0.33**	–	–	–	2.72 (0.64)	−3.37**	0.44
Mentally ill/unstable	–	–	–	3.16 (0.73)	1.71	0.23	3.14 (0.72)	1.54	0.20
Narcissism	3.37 (1.53)	−3.12**	0.41	4.23 (1.52)	1.14	0.15	3.73 (1.86)	−1.12	0.15
Psychopathy	**4.90 (1.69)**	4.07***	**0.53**	**5.40 (1.13)**	9.41***	**1.25**	**4.84 (1.41)**	4.54***	**0.59**
Machiavellianism	**5.51 (1.51)**	7.61***	**1.00**	4.35 (1.71)	1.55	0.21	3.66 (1.66)	−1.57	0.20
Punishment									
Warning	1.21 (0.70)	−30.62***	4.02	1.26 (0.90)	−23.04***	3.05	1.93 (1.43)	−11.06***	1.45
Fine	1.72 (1.51)	−11.50***	1.51	1.98 (1.67)	−9.10***	1.21	2.71 (1.89)	−5.21***	0.68
Suspended sentence	2.64 (2.01)	−5.17***	0.68	2.96 (1.96)	−4.00***	0.53	3.97 (1.82)	−0.15	0.02
Temporal sentence	**5.33 (1.95)**	5.19***	**0.68**	**5.51 (1.94)**	5.88***	**0.78**	**5.33 (1.72)**	5.88***	**0.77**
Life sentence	4.38 (2.22)	1.26	0.17	3.64 (2.14)	−1.25	0.17	2.59 (1.76)	−6.01***	0.80

Variables between ‘rape’ and ‘mentally ill/unstable’ were rated on a 5-point scale; variables between ‘narcissism’ and ‘life sentence’ were rated on a 7-point scale. Missings indicate variables with insufficient interrater reliabilities. *p < 0.05, **p < 0.01, ***p < 0.001. Values in bold represent values that are significantly greater than the middle scale points (i.e., participants experienced these as especially likely or appropriate).

##### 3.1.2.2 Offender

In an open text field, participants were asked to briefly describe the offender they imagined. For example, one participant’s answer was: ‘Person with pedophilic inclination with no scruples or morals.’ These answers were coded by two blind raters to determine the extent to which they matched certain categories. Specifically, the raters rated the participants’ responses along the following two categories on a coding scale ranging from 1 = *does not match at all* to 5 = *absolutely matches*: pedophile (ICC_SC_ = 0.74, ICC_SV_ = −0.14, ICC_CV_ = 0.57), mentally ill/unstable (ICC_SC_ = 0.15, ICC_SV_ = 0.68, ICC_CV_ = 0.69). Here too, most categories achieved satisfactory inter-rater reliabilities with an ICC of above 0.30. The categories that did not result in sufficient inter-rater reliabilities were excluded from further analyses (see Table 1 for included and excluded variables).

In order to capture more specific aspects of the imagined offender personality, participants also answered the Dirty Dozen short scale with nine items to measure the ‘Dark Triad’ (Küfner et al., 2014). It should be borne in mind that this scale is actually a self-report measure and was not designed to assess imagined others. However, since we were not interested in a valid assessment of a third party, but rather in the implicit theories of our participants, we considered this scale to be suitable. Participants completed three items from the narcissism subscale (e.g., ‘The offender tends to wish to be admired by others’; α_SC_ = 0.85, α_SV_ = 82, α_CV_ = 0.92), the psychopathy subscale (e.g., ‘The offender tends to have no remorse’; α_SC_ = 0.88, α_SV_ = 0.58, α_CV_ = 0.76), and Machiavellianism subscale (e.g., ‘The offender tends to manipulate others to have his or her will’; α_SC_ = 0.82, α_SV_ = 0.84, α_CV_ = 0.80) on a scale from 1 = *not at all* to 7 = *very much*.

##### 3.1.2.3 Punishment

Participants were asked to indicate how they would prefer to punish the offender on a predefined scale adapted from previous research on attitudes towards punishment (Häßler and Greve, 2012; Suhling et al., 2005; Tremblay, 1988). They rated the 5 items ‘warning,’ ‘fine,’ ‘suspended sentence,’ ‘temporal sentence,’ and ‘life sentence’ on a 1 = *not at all appropriate* to 7 = *very appropriate* scale.

### 3.2 Results

To test whether people associated specific offenses, offenders, and punishments with the keywords, we applied one sample t-tests on the variables (generated either by the raters’ scores on the participants’ open responses or participants’ responses on the scales) for each keyword pair. In these tests, we investigated whether the sample mean was statistically different from the respective mean scale point, which reflects a neutral assessment and was chosen when participants had not indicated any particular idea about the keywords (neither perceived as unlikely nor likely). Significant deviations from the mean scale points thus indicated that the participants had systematic schemas for the respective keywords. For descriptive statistics and results of these t-tests, see Table 1.

### 3.3 Discussion

Results showed that keywords containing ‘sex’ activated certain schemas of criminal cases in the participants’ minds. In particular, we found that participants associated the combined keywords ‘sex and children’ not only with sexual abuse, but also with physical violence. The offender was classified as pedophile, psychopathic and Machiavellian with above-average frequency. The combined keywords ‘sex and violence’ were associated with a wider range of offenses (rape and physical violence as well as coercion, harassment, or insult) and comparatively fewer negative impressions of the offender (psychopathic). In contrast, the combined keyword pair ‘children and violence’ triggered heterogenous associations: The participants found both physical and psychological acts of violence as possible offenses, and psychopathy likely to describe the personality of the offender. While a temporal sentence was perceived as an appropriate punishment in the context of all keyword pairs, a life sentence was only indicated as particularly inappropriate for ‘children and violence.’

Interestingly, the pattern of Study 1 matched the results of the Pilot Study, quantifying its findings. To avoid influencing participants with predefined offenses and offenders, we used mostly open text fields. However, some variables were measured using predefined scales, such as the Dirty Dozen scale (a scale that is not unquestioned, especially with regard to the construct of narcissism, Kajonius et al., 2016, but also see Tommasi et al., 2025). But these were placed after participants had formed their own opinions about the case, so we are confident that they did not influence their responses. However, the open-ended response options had the limitation that some of the participants’ responses were not clear enough for our raters, which lead to insufficient interrater reliabilities and prevented us from evaluating all response options. This limited the informative value and was to be remedied in the subsequent study.

## 4 Study 2

The previous studies showed that it does not take much information to trigger specific negative schemas in relation to sexual crimes. In Study 2, we aimed to investigate whether these negative schemas bias impression formation about a criminal case. Therefore, participants were asked to read a superficial report about a court case that contained one of the keyword pairs. Varying a single attribute in an unaltered vignette is an approach that has been used in a similar way in other studies on the halo effect (e.g., Forgas, 2011). Furthermore, we improved the methodology in three ways: (1) To avoid ambiguous responses, we eliminated open text fields and predefined all variables using standardized scales that measured the offenses, offenders, and punishments imagined by participants. Due to the use of standardized scales, in Study 2 we also chose to survey people who had no prior training in criminal terminology. In this way, we sought to map the response behavior of the general population, and took into account a possible ceiling effect. (2) We used a more conservative between-subjects design instead of a within-subjects design. (3) We investigated whether the critical keywords only elicited a devil effect when they were paired. Therefore, participants were not only presented with the critical keyword pairs, but also keywords paired with a neutral filler word. We decided to use ‘trees’ as filler word because we considered that it is neither negatively nor positively charged and not associated with a criminal act per se.

We expected that the combination of the keywords ‘sex and children’ would bias the impression of the criminal case according to a schema of violence, pedophilia, and harsh punishment. Similarly, we hypothesized that the combination of the keywords ‘sex and violence’ would influence the impression of the case according to a schema with a violent and mentally ill offender and maximum sentences. We expected no systematic bias for the combined keywords ‘children and violence’ and for the single keywords in combination with the filler word.

### 4.1 Method

#### 4.1.1 Participants and design

The sample consisted of 236 participants (164 female, 71 male, 1 did not specify gender; *M* = 29.08 years, *SD* = 11.32) who voluntarily participated in the study. They were recruited via German social media websites and on the platform of an Austrian university; the latter could receive research credit for participation. It is noteworthy that only some of our participants received extrinsic compensation in the form of research credit. However, these were in the position to choose from a range of studies and explicitly opted for this study, which is why we assumed that this did not cause any major differences in motivation compared to people without extrinsic compensation.

Of the complete sample, *n* = 140 stated that they were studying (psychology: *n* = 119, art/culture/languages: *n* = 6, medicine: *n* = 4, education/teaching/nursing: *n* = 3, engineering/IT: *n* = 3, geology/chemistry/physics: *n* = 2, business: *n* = 2, not specified: *n* = 1), *n* = 79 stated that they were working (socio-educational field: *n* = 13, medicine/pharmacy: *n* = 7, business: *n* = 5, craftsman/IT/engineering/physics: *n* = 4, art/journalism: *n* = 2, service workers: *n* = 2, not specified: *n* = 46), and *n* = 17 stated ‘other’ to the question of their current status (parental leave/housewife: *n* = 5, retired: *n* = 3, school: *n* = 2, voluntary work: *n* = 1, not specified: *n* = 6). Notably, German and Austrian psychology studies do not usually include forensic content, which is why we did not assume that the rather large number of psychology students had a high level of expertise on the subject of sexual offenses.

The study followed a between-subjects design with random assignment to the seven conditions in which the keyword pairs varied (sex and children vs. sex and violence vs. children and violence vs. trees and sex vs. trees and children vs. trees and violence vs. no keywords); the variables covering imagined offenses, offenders, and punishments served as dependent variables.

#### 4.1.2 Procedure and materials

The study was conducted in the form of an online survey. After informed consent was obtained, participants were presented with the following report:

‘Today, the trail for the recently released W. case began at Nuremberg Regional Court. The opening of the trial was followed with great interest by the press. The case was also the subject of public discussion. There was a flurry of flashbulbs as W. left the car. At the beginning of the proceedings, W.’s defendant announced that he would not comment on the case, motives or the background today. The lawyer described the accusations as absurd and questioned the credibility of the evidence. He also emphasized that the case should be dealt with by the court and not by the media. After several motions for evidence were made, the hearing was adjourned. In the following sessions, witnesses and experts will be heard one after another. We will report further.’

This text was headed: ‘Court verdict in the W. case eagerly awaited.’ Importantly, we also included a keyword pair in the title to represent the seven different conditions. This was, for example: ‘Sex and children: Court verdict in the W. case eagerly awaited’ or ‘Trees and sex: Court verdict in the W. case eagerly awaited.’ No keywords were added in the seventh condition. After reading the text, participants answered questions about their ideas about the offense, the offender, and the punishment they perceived they felt was appropriate. At the end, the participants were thanked and debriefed as in Study 1. The study was conducted in German. It was approved by the Ethics Council of the Institute of Psychology at the University of Klagenfurt (ER-PSY; ethics ID reference number: 2018–082).

##### 4.1.2.1 Offense

Participants were asked to indicate on a predetermined scale which offenses they considered likely in connection with the text they had read. They rated possible offenses using items taken from the German Criminal Code on response scales ranging from 1 = *very unlikely* to 10 = *very likely*. In addition to the variables collected in Study 1 (rape, child sexual abuse, coercion/harassment/insult, physical violence, psychological violence, forced prostitution, child abduction), the participants answered additional pseudo items that served to conceal the aim of the study (corruption, drunk-driving, robbery, extortion, misuse of checks and credit cards, non-assistance, pimping, murder).

##### 4.1.2.2 Offender

Participants were asked to indicate how likely (1 = *very unlikely* to 10 = *very likely*) they experienced the following three statements regarding the offender: ‘W. is pedophile,’ ‘W is mentally ill,’ ‘W. is dangerous.’

##### 4.1.2.3 Punishment

Finally, participants decided which punishment they considered appropriate for the accused person. We modified the categories from Study 1 by removing the less relevant ‘warning’ and adding finer categories of temporal sentences as well as the death penalty (which is not part of the judicial system in almost all European countries but was mentioned by participants in the Pilot Study). The participants rated the following items on a scale from 1 = *not at all appropriate* to 10 = *very appropriate*: fine, suspended sentence, temporal sentence (6 months), temporal sentence (8 years), temporal sentence (15 years), death penalty. Moreover, participants answered additional pseudo items that served to disguise the aim of the study (charitable contribution, social service, mediation between victim and offender, financial compensation).

### 4.2 Results

To investigate whether people associated specific offenses, offenders, and punishments with the report as a function of title, we applied 2 (sex vs. no sex) × 2 (children vs. no children) × 2 (violence vs. no violence) ANOVAs on the variables. Notably, due to our design, we could only compare combinations of two words. Significant interaction effects indicated that the participants’ judgments were biased by systematic schemas activated by the critical keyword pairs (compared to when one of the keywords was missing). For descriptive statistics, see Table 2, for full results of all ANOVAs performed, see Table 3. In the following, we only report significant interaction effects and break down the latter.

**Table 2:** Means and standard deviations (in parentheses) of participants’ assessments (Study 2).

	Sex and children (*n* = 38)	Sex and violence (*n* = 34)	Children and violence (*n* = 32)	Trees and sex (*n* = 32)	Trees and children (*n* = 35)	Trees and violence (n = 32)	No keywords (n = 33)
Offense							
Rape	6.76 (2.64)	6.32 (2.80)	5.94 (2.77)	5.10 (2.40)	4.82 (2.42)	4.41 (2.43)	4.79 (2.48)
Child sexual abuse	6.71 (2.58)	4.68 (2.66)	6.09 (2.83)	4.81 (2.76)	4.09 (2.32)	4.16 (2.55)	4.00 (2.49)
Coercion/harassment/insult	7.00 (2.38)	6.59 (2.65)	6.03 (2.79)	5.55 (2.58)	5.06 (2.28)	4.16 (2.20)	4.82 (2.46)
Physical violence	5.66 (2.92)	6.65 (2.49)	6.19 (2.57)	4.74 (2.38)	5.33 (2.72)	4.78 (2.60)	4.88 (2.07)
Psychological violence	5.58 (2.94)	5.74 (2.35)	6.41 (2.46)	4.71 (2.21)	5.52 (2.65)	4.72 (2.37)	4.39 (2.47)
Forced prostitution	3.55 (2.44)	3.50 (2.49)	3.66 (2.28)	3.74 (2.19)	3.52 (1.81)	3.38 (2.21)	3.70 (2.28)
Child abduction	4.79 (3.09)	3.97 (2.63)	4.28 (2.48)	3.74 (2.31)	4.42 (2.21)	4.38 (2.50)	3.82 (2.23)
Offender							
Pedophile	5.71 (2.59)	4.03 (2.62)	5.41 (2.55)	4.22 (2.31)	3.63 (2.29)	3.62 (2.12)	3.21 (2.00)
Mentally ill	5.13 (2.61)	4.91 (2.47)	5.31 (1.98)	4.88 (2.14)	4.57 (2.08)	4.19 (2.25)	3.79 (1.88)
Dangerous	6.13 (2.22)	6.12 (2.28)	5.91 (1.98)	4.94 (2.09)	5.29 (1.98)	5.19 (2.33)	4.70 (2.42)
Punishment							
Fine	4.24 (2.71)	4.64 (2.78)	4.60 (3.13)	4.74 (2.29)	4.82 (2.56)	4.87 (2.86)	5.18 (2.37)
Suspended sentence	3.39 (2.52)	4.27 (2.92)	3.97 (2.40)	3.84 (2.58)	4.45 (1.99)	4.27 (2.65)	4.45 (2.15)
Temporal sentence (6 months)	4.32 (2.60)	4.61 (2.59)	4.93 (2.38)	3.87 (2.11)	4.12 (2.30)	4.47 (2.78)	4.91 (2.23)
Temporal sentence (8 years)	5.11 (2.81)	5.58 (2.31)	5.13 (2.53)	4.19 (2.32)	4.21 (2.70)	5.07 (2.70)	5.12 (2.43)
Temporal sentence (15 years)	4.79 (2.89)	5.61 (2.63)	5.13 (2.81)	4.32 (2.95)	4.06 (2.81)	5.17 (2.96)	5.15 (2.91)
Death penalty	1.61 (1.65)	2.18 (2.44)	2.13 (2.57)	1.68 (1.38)	2.03 (1.81)	2.33 (2.38)	1.70 (1.51)

All variables were rated on a 10-point scale (1 = very unlikely/not at all appropriate to 10 = very likely/very appropriate).

**Table 3:** Results of 2 (sex vs. no sex) × 2 (children vs. no children) × 2 (violence vs. no violence) ANOVAs (Study 2).

	Main effect of sex		Main effect of children		Main effect of violence		Sex × children interaction		Sex × violence interaction		Children × violence interaction	
	*F*	η_p_^2^	*F*	η_p_^2^	*F*	η_p_^2^	*F*	η_p_^2^	*F*	η_p_^2^	*F*	η_p_^2^
Offense												
Rape	18.35***	**0.08**	11.45**	**0.05**	5.07*	**0.02**	3.40	0.02	3.17	0.01	2.77	0.01
Child sexual abuse	13.95***	**0.06**	16.39***	**0.07**	4.06*	**0.02**	4.08*	**0.02**	0.10	<0.001	4.09*	**0.02**
Coercion/harassment/insult	27.41***	**0.11**	14.17***	**0.06**	3.30	0.01	1.99	0.01	3.82	0.02	3.52	0.02
Physical violence	5.18*	**0.02**	7.30**	**0.03**	8.11**	**0.03**	0.27	0.001	4.98*	**0.02**	1.13	0.01
Psychological violence	1.82	0.01	11.27**	**0.05**	4.93*	**0.02**	0.08	<0.001	0.63	0.003	0.41	0.002
Forced prostitution	0.05	<0.001	<0.001	<0.001	0.10	<0.001	<0.001	<0.001	0.01	<0.001	0.34	0.002
Child abduction	0.01	<0.001	1.82	0.01	0.15	0.001	0.26	0.001	0.14	0.001	0.62	0.003
Offender												
Pedophile	12.15**	**0.05**	16.00***	**0.07**	4.22*	**0.02**	1.77	0.01	0.53	0.002	2.73	0.01
Mentally ill	5.16*	**0.02**	4.72*	**0.02**	1.42	0.01	0.48	0.002	0.22	0.001	0.19	0.001
Dangerous	5.73*	**0.02**	7.54**	**0.03**	6.43*	**0.03**	0.66	0.003	0.81	0.004	0.03	<0.001
Punishment												
Fine	1.09	0.01	0.90	0.004	0.23	0.001	0.02	<0.001	0.05	<0.001	0.01	<0.001
Suspended sentence	2.23	0.01	0.64	0.003	0.03	<0.001	0.27	0.001	0.50	0.002	0.12	0.001
Temporal sentence (6 months)	0.04	<0.001	0.32	0.001	2.08	0.01	2.15	0.01	1.85	0.01	2.08	0.01
Temporal sentence (8 years)	1.00	0.004	0.30	0.001	5.58*	**0.02**	4.26*	**0.02**	2.51	0.01	1.15	0.01
Temporal sentence (15 years)	0.51	0.002	0.01	<0.001	4.84*	**0.02**	2.51	0.01	1.57	0.01	1.08	0.01
Death penalty	0.63	0.003	0.02	<0.001	1.51	0.01	0.35	0.002	0.04	<0.001	0.56	0.003

*p < 0.05, **p < 0.01, ***p < 0.001. Values in bold represent significant effects.

Notably, the following analyses cover the variables which served to test the hypotheses. However, the pseudo items were also examined for the sake of completeness. These analyses can be found in the Online Supplementary (https://doi.org/10.17605/OSF.IO/MUQTH).

**Figure 1:**
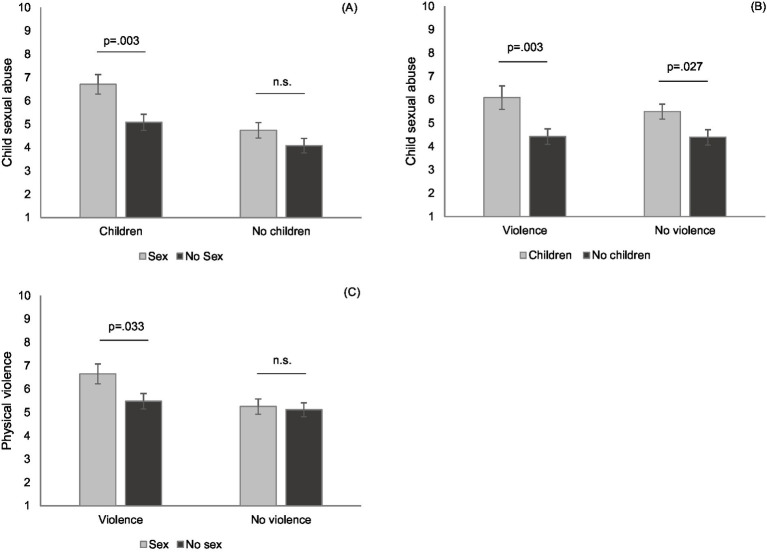
The influence of different keyword pairs on the participants’ perceived likelihood that child sexual abuse **(A,B)** or physical violence **(C)** is associated with the crime in question (Study 2); error bars represent ± 1 SE.

The ANOVA on child sexual abuse showed a significant sex × children interaction effect, *F*(1,226) = 4.08, *p* = 0.045, η_p_^2^ = 0.02, 95%CI = [0.00, 0.07]. Simple main effect analyses revealed that when the keyword ‘children’ was absent, participants rated it similarly likely or unlikely that the case was about sexual abuse regardless of whether they read ‘sex’ in the title or not, *F*(1,226) = 2.11, *p* = 0.148, η_p_^2^ = 0.01, 95%CI = [0.00, 0.05]. However, when the keyword ‘children’ was present, participants were more likely to categorize the case as sexual abuse when it was combined with ‘sex’ (vs. no ‘sex’), *F*(1,226) = 9.28, *p* = 0.003, η_p_^2^ = 0.04, 95%CI = [0.005, 0.10], see Figure 1A. This was not surprising, but demonstrated an effective manipulation of the title words.

The ANOVA on child sexual abuse revealed a significant children × violence interaction effect, *F*(1,226) = 4.09, *p* = 0.044, η_p_^2^ = 0.02, 95%CI = [0.00, 0.07]. Simple main effect analyses showed that participants were more likely to rate the case as sexual abuse when the keyword pair was combined with ‘children’ (vs. ‘no children’), both when ‘violence’ was present, *F*(1,226) = 8.96, *p* = 0.003, η_p_^2^ = 0.04, 95%CI = [0.005, 0.10], as well as when it was not present, *F*(1,226) = 4.93, *p* = 0.027, η_p_^2^ = 0.02, 95%CI = [0.00, 0.07], see Figure 1B. Remarkably, the former effect was larger, indicating a greater plausibility for a more violent plot for child sexual abuse.

The ANOVA on temporal sentence (8 years) showed a significant sex × children interaction effect, *F*(1,221) = 4.26, *p* = 0.040, η_p_^2^ = 0.02, 95%CI = [0.00, 0.07]. Descriptive statistics indicated that when the keyword ‘children’ was absent, participants experienced a temporal sentence of 8 years to be equally appropriate or inappropriate, regardless of whether they read ‘sex’ in the title or not. However, when the keyword ‘children’ was present, participants perceived such a temporal sentence as more appropriate when combined with ‘sex’ (as opposed to no ‘sex’). Thus, a severe punishment was seen as particularly appropriate for child sexual abuse. However, simple main effect analyses revealed no statistical differences between these conditions, all *p*s ≥ 0.356. Notably, this pattern was statistically significantly reflected in the pseudo item ‘charitable contribution,’ which is outlined in the Online Supplementary. According to this, a punishment in the form of a charitable contribution was perceived as particularly inappropriate when they keyword ‘children’ was coupled with ‘sex.’

Finally, the ANOVA on physical violence revealed a significant sex × violence interaction effect, *F*(1,226) = 4.98, *p* = 0.027, η_p_^2^ = 0.02, 95%CI = [0.00, 0.07]. Simple main effect analyses revealed that when the keyword ‘violence’ was absent, participants were similarly likely or unlikely to believe that the case involved physical violence, regardless of whether or not they read ‘sex’ in the title, *F*(1,226) = 0.05, *p* = 0.832, η_p_^2^ < 0.001, 95%CI = [0.00, 0.01]. However, when the keyword ‘violence’ was present, participants were more likely to associate the case with physical violence when it was combined with ‘sex’ (compared to no ‘sex’), *F*(1,226) = 4.59, *p* = 0.033, η_p_^2^ = 0.02, 95%CI = [0.00, 0.07], see Figure 1c. In other words, because they experienced physical violence as particularly likely when a crime involved sex, participants suspected a more brutal act for rape than for other assaults.

### 4.3 Discussion

Study 2 yielded several insights. First, we were able to find further evidence for the devil effect triggered by sexual crimes. Specifically, child sexual abuse was rated as particularly likely when the keyword pair included not only children but also violence. This points to the devil effect, as child sexual abuse is coupled with violence in the perception of the participants. Further, participants found physical violence to be particularly likely when violence was combined with sex. This also highlights the devil effect, as sexual offenses, in contrast to other offenses, were particularly violent in the subjects’ perceptions. Study 2 thus supports the assumption that keyword pairs containing ‘sex’ activate schemas of criminal cases (especially in the form of particularly violent offenses) that bias subsequent impression formation.

As a second insight, Study 2 showed that the keywords sex, children, and violence did not produce a devil effect when they were not paired with each other. Instead, they elicited associations with a wide range of both low- and high-threshold offenses (see main effects in Table 3). For example, the keyword ‘sex’ triggered associations with coercion, harassment, and insult but also with rape, child sexual abuse, and physical violence. (When interpreting the main effects, it should be noted that these represent the mean value of all combinations – regardless of whether they were combined with one of the critical words or our filler word ‘trees.’ Investigating the effects of the single keywords was not possible due to the methodological design, as the words ‘sex,’ ‘children,’ and ‘violence’ were not presented as standalones in the news title, but were combined either with each other or with the filler word.) Notably, these effects were particularly strong. Especially the main effect of sex was stronger than the effect of the keyword pairs.

Despite these consistent results, some limitations of the current design should be considered. To investigate whether our keywords only evoked a devil effect when paired, we used the filler word ‘trees’ as it did not appear to be negatively or positively charged and was not associated with a criminal act per se. However, we cannot rule out the possibility that the word ‘trees’ caused confusion among the participants, *because* it was not associated with a criminal offense. Moreover, it should be noted that, similar to Study 1, the critical word pairs did not affect every variable examined, but only some of them. This suggests that the devil effect triggered by sexual crimes is not so strong that it overshadows all information, but that it is still strong enough to activate some preconceived notions. It also indicates that there was no ceiling effect, even though no informed sample was examined in this study.

## 5 General discussion

The present studies provided convergent evidence of a devil effect triggered by sexual crimes. This means that mere keywords that include ‘sex’ trigger specific negative schemas which bias the impression of criminal cases.

Specifically, in both the Pilot Study and Study 1, the combination of the keywords ‘sex and children’ activated an extremely negative schema of criminal cases which included acts of violence, pedophile offenders, and the desire for harsh punishments. The combined keywords ‘sex and violence’ triggered more realistic impressions of offenses, but still very negative impressions of possible offenders, and maximum sentences. In contrast to these extreme impressions were the rather heterogenous reactions to the combined keywords ‘children and violence.’ Study 2 also showed that these negative schemas guided the impression of a criminal case about which the readers were only superficially informed. It also revealed that the devil effect only occurred when the critical keywords were combined.

### 5.1 The results in the context of earlier research

These findings support the idea of the devil effect (Thorndike, 1920) and transfer it to a new context. It is already known that various attributes, from personal names (Lebuda and Karwowski, 2013) to temporary signals such as smiles (Forgas et al., 1983), can produce halo or devil effects. The devil effects triggered by sexual crimes can be included in this range. A number of stereotypical beliefs about sexual offending have already been identified in past research (e.g., Fuselier et al., 2002; Horvath and Brown, 2009). However, the current work is the first to show that these stigmatizing judgments may be the result of a devil effect.

How do (halo and) devil effects occur in practice? It is assumed that automatic and constructive Gestalt processes take place that combine and reinterpret all available information into a coherent whole (Asch, 1946; Kelley, 1950). That people piece together a coherent whole from the information they have has also been suggested in other work: Bruner and Tagiuri (1954) proposed that people assume that traits are interrelated, for example, that an intelligent person is also creative. They called this concept implicit personality theory. Hamilton and Rose (1980) noted that people often perceive a relationship between variables even when no such relationship exists. They referred to this concept as illusory correlation. In our context, similar processes could take place: As soon as the keyword pairs ‘sex and children’ or ‘sex and violence’ are available, automatic processing could be activated that reflexively interprets all information according to predefined categories to create a coherent whole. Notably, the halo effect appears so robust that people remain susceptible to it even when asked to reflect on their cognitive processes or the halo effect itself (Wetzel et al., 1981). Thus, the devil effect triggered by sexual crimes is probably quite pervasive.

### 5.2 Implications of the current findings

It is known that judges and juries are influenced by biases, such as the anchoring effect, where people rely too much on the first piece of information offered (Englich et al., 2006). The devil effect triggered by sexual crimes could further distort judgment. An argument against a strong devil effect in the justice system is that the conviction rate for sexual assault and child sexual abuse that go to court is very low (e.g., Walinchus et al., 2025). In the justice system, a case is usually dealt with at several levels (from the police to the court). In the final stage, judges and juries read all the evidence, carry out a meticulous evidential process, and have to justify their decision. It seems possible that the devil effect could be mitigated by this intensive information processing. However, this consideration is contradicted by findings that show that jurors’ verdicts in sexual assault trials can still be biased even after they have painstakingly processed the evidence (Nitschke et al., 2022).

The devil effect triggered by sexual crimes could also contribute to an increase in the fear of crime in society (e.g., Deitert, 2012) and public demand for harsher punishments, even though these are known to be unable to prevent such crimes (e.g., Cullen et al., 2011). This could also be a cyclical relationship: On the one hand, the devil effect could contribute to the fear of crime; on the other hand, a corresponding fear could further strengthen the devil effect. Furthermore, the devil effect could have a negative impact on social rehabilitation and even therapy for offenders. This is supported by the fact that many psychotherapists and those in training are not willing to work with people who are associated with such crimes; in fact, some of them justify this decision with a negative attitude towards these people (Jahnke et al., 2015b; Stiels-Glenn, 2010).

Another implication of the current findings concerns the public safety perspective. Preconceived opinions about sexual crimes may impair perceptions about how these offenses are most commonly committed. By assuming that sexual crimes are usually committed by people who are violent, mentally ill, or have a disruptive personality, people may not look more closely at actual offenders. In addition, victims may feel that they are not believed because their case does not fit the mold. Overcoming the devil effect could therefore raise people’s awareness and lead to a better protection for potential victims.

As described above, the devil effect is extremely reliable and pervasive; even explicit interventions and instructions cannot eliminate it (Wetzel et al., 1981). However, it can be reduced by a more systematic and attentive information processing (Sigall and Ostrove, 1975). To avoid the devil effect, it would therefore make sense to use procedures that motivate a systematic information processing strategy. This could be done, for example, by using a devil’s advocate by default prior to the adjudication. The devil’s advocate is defined as a person who takes a critical look at the other side of an issue. The purpose of this idea is to evaluate the quality of the original thought and to recognize flaws in the own reasoning. In a study by Lidén et al. (2019), prosecutors were even able to act as their own devil’s advocate, influencing their assessment prior to indictment. Other research makes recommendations specifically for judges to encourage more systematic information processing, e.g., through the use of formal protocols, longer decision times, or discussions with others (Casey et al., 2013). However, not only among judges and jurors, but also in political dialogues and media coverage of sexual offenses, strategies should be used to create dissent and get people to look at a case more closely in order to overcome the devil effect triggered by sexual crimes in the broader society as well. This would enhance an optimal handling of the devil effect – which, of course, may be utopian to some extent, because there are also motives in politics and the media to dramatize information for the own benefit (e.g., for repressive policies or public attention; Dowler, 2006).

### 5.3 Limitations and future research

The current research benefited from a mixed methods design that compensated for the shortcomings of the individual approaches, as well as a range of materials that were improved over the course of the studies. Revealing a consistent pattern, it produced replicable results. Nevertheless, some limitations must be noted.

On the one hand, the limitations concern our sample: We examined different groups of people across all studies: While in the Pilot Study, it was professionals from a healthcare company and students of different study programs, in Study 1, it was law students, and in Study 2, a mixed group of people. We therefore had people with different professional backgrounds and different levels of expertise. All of these showed the devil effect, which leads us to believe in the robustness of the effect. The findings could plausibly be transferred to a jury panel, which is usually randomly selected. Study 1 in particular suggests that the findings could also be transferred to more experienced people in the field of law. However, whether the findings can really be transferred to specialists such as judges and prosecutors, who are familiar with sexual offenses in detail and may also have developed their own strategies for systematic information processing, is an open question.

On the other hand, the limitations concern our operationalization: We used compact stimuli consisting of only two keywords to activate the devil effect. The fact that we found an effect despite this brevity supports our reasoning. However, in real criminal cases, more information is usually accessible, and recent findings show that abstract vs. concrete information can elicit different responses when it comes to assessing sexual offenses (Smith et al., 2022). Thus, it would be beneficial for future research to investigate how more information could distort or correct impression formation. Further, in our studies, the keyword pairs sequence was always such that we placed the word ‘sex’ first. It cannot be ruled out that this produced an anchoring effect. Thus, future studies should ensure that also the order of words is randomized. Lastly, we used a control condition in the form of the keywords ‘children and violence’ to distinguish the effects of sexual crimes from those of other crimes. Importantly, we did not find a devil effect activated by this, although the underlying crime was certainly considered morally wrong by most participants. Nevertheless, it would be worthwhile to extend the control condition to investigate whether sexual crimes are indeed unique in their appearance or comparable to certain other crimes. It should also be borne in mind that there are cultures in which not only sexual crimes evoke a devil effect, but in which ‘children and violence’ also activate specific schemas (see, for example, the so-called moral panic about youth crime in North America; Silcox, 2022). Cultural aspects such as these must therefore be carefully considered in future studies.

Future research would benefit from investigating possible boundary conditions. For example, the halo effect induced by physical attractiveness does not work or is even counterproductive when a person relies on their attractiveness to commit an antisocial act such as swindle (Sigall and Ostrove, 1975). It would be worthwhile to investigate whether the devil effect triggered by sexual crimes is also limited by certain conditions. Finally, it would be helpful to investigate why the devil effect exists as such. One possible reason could be the fear of crime, which could feed the schemas underlying the devil effect exist. The schemas might also be socialized by corresponding media coverage, which is known to be angrier and more emotionally negative in the case of sexual crimes (Harper and Hogue, 2015). Another reason for the existence of the devil effect could be that sexual offenses violate social norms to protect women and children and that people are eager to signal their social responsibility by loudly condemning them.

## 6 Conclusion

If you read a newspaper article with the headline ‘Sex and children: Court verdict eagerly awaited,’ you might be surprised not to read about a gruesome case of a pedophile sex offender. The current work provides an answer as to why this is the case: due to a devil effect triggered by sexual crimes. This effect could lie behind our seemingly built-in fear and loathing of sexual crimes and could have serious consequences, from reduced awareness of actual crimes to biased judgments by judges and juries.

## Data Availability

The datasets presented in this study can be found in online repositories. The names of the repository/repositories and accession number(s) can be found below: https://doi.org/10.17605/OSF.IO/MUQTH.
